# Essential Role of Adhesion GPCR, GPR123, for Human Pluripotent Stem Cells and Reprogramming towards Pluripotency

**DOI:** 10.3390/cells12020304

**Published:** 2023-01-13

**Authors:** Olga A. Krasnova, Karina A. Kulakova, Julia V. Sopova, Evgenyi Y. Smirnov, Sergey A. Silonov, Ekaterina V. Lomert, Olga A. Bystrova, Marina G. Martynova, Irina E. Neganova

**Affiliations:** 1 Laboratory of Molecular Medicine, Institute of Cytology of the Russian Academy of Sciences, Tikhoretsky Ave. 4, 194064 St-Petersburg, Russia; 2 Center of Transgenesis and Genome Editing, St. Petersburg State University, Universitetskaja Emb., 7/9, 199034 St-Petersburg, Russia; 3 Laboratory of Regulation of Genes Function, Institute of Cytology of the Russian Academy of Sciences, Tikhoretsky Ave. 4, 194064 St-Petersburg, Russia; 4 Laboratory of Structural Dynamics, Stability and Folding of Proteins, Institute of Cytology of the Russian Academy of Sciences, Tikhoretsky Ave. 4, 194064 St-Petersburg, Russia; 5 Laboratory of Molecular Genetics of Tumor Cells, Institute of Cytology of the Russian Academy of Sciences, Tikhoretsky Ave. 4, 194064 St-Petersburg, Russia; 6 Laboratory of Cell Morphology, Institute of Cytology of the Russian Academy of Sciences, Tikhoretsky Ave. 4, 194064 St-Petersburg, Russia

**Keywords:** human pluripotent stem cells, pluripotency, reprogramming, GPCRs, G proteins, adhesion GPCRs, GPR123

## Abstract

G-protein-coupled receptors (GPCRs) are the largest family of cell surface receptors. They modulate key physiological functions and are required in diverse developmental processes including embryogenesis, but their role in pluripotency maintenance and acquisition during the reprogramming towards hiPSCs draws little attention. Meanwhile, it is known that more than 106 GPCRs are overexpressed in human pluripotent stem cells (hPSCs). Previously, to identify novel effectors of reprogramming, we performed a high-throughput RNA interference (RNAi) screening assay and identified adhesion GPCR, GPR123, as a potential reprogramming effector. Its role has not been explored before. Herein, by employing *GPR123* RNAi we addressed the role of GPR123 for hPSCs. The suppression of *GPR123* in hPSCs leads to the loss of pluripotency and differentiation, impacted colony morphology, accumulation of cells at the G2 phase of the cell cycle, and absence of the scratch closure. Application of the *GPR123* RNAi at the initiation stage of reprogramming leads to a decrease in the percentage of the “true” hiPSC colonies, a drop in E-cadherin expression, a decrease in the percentage of NANOG+ nuclei, and the absence of actin cytoskeleton remodeling. Together this leads to the absence of the alkaline-phosphatase-positive hiPSCs colonies on the 18th day of the reprogramming process. Overall, these data indicate for the first time the essential role of GPR123 in the maintenance and acquisition of pluripotency.

## 1. Introduction

Currently, reprogramming technology for generating human-induced pluripotent stem cells (hiPSCs) has become widespread. These cells possess very similar characteristics to human embryonic stem cells (hESCs) and are widely used in research modeling of various human diseases, drug testing, and as a source of cells for regenerative medicine, for example, for autologous cell therapies. At the same time, our knowledge of the factors and signaling cascades that play an important role in the induction and maintenance of pluripotency is still very limited.

Previously, in searching for the new signaling molecules, which are important for hiPSCs generation, we used a high-throughput small interfering RNA (RNAi) screening assay during the initiation phase of reprogramming. We performed specific knockdown of 784 members of the different kinases and phosphatases from the Dharmacon library and revealed 6 members of the G-protein-coupled receptors (GPCRs) expected to be important for reprogramming [1]. Adhesion GPR123 was identified by this screen as a potential effector for the reprogramming process and this was the rationale for the present study. Importantly, this is the first study which addresses in detail the role of GPR123 for hPSCs and in the reprogramming process during hiPSCs generation.

Adhesion GPCRs is a class of 33 human protein receptors, but despite their broad distribution and modern screening techniques, 17 of them are still without known ligands and most of these proteins are orphans [2,3,4]. Adhesion GPCRs are known to be expressed in white blood cells, neurons, embryonic cells, reproductive tract cells, and various tumors. They are important in homeostasis, induction of PKA activity, c-AMP binding, activation of insulin signaling, and signaling of NOD-like receptors [4].

Regrettably, the role of adhesion GPCRs is practically unknown or poorly studied for stem cells. At the same time, according to Nakamura and colleagues [5], the *EDG5*, *GPR20*, and *GPR123* genes have a significantly higher expression level in hESCs compared with 100 types of somatic cells studied. Moreover, GPR125, which is a group III adhesion receptor, like GPR123, has been shown to be involved in maintaining pluripotency of stem cells and is a known germ line precursor marker [6]. GPR123 together with GPR124 and GPR125 form a separate phylogenetic group among the adhesion GPCRs [7]. It is important to note that, in its primary structure, GPR123 differs from the other members of the adhesion GPCRs. The functional specificity of these receptors is largely determined by the presence of a long extracellular N-terminal fragment (NTF) containing conserved protein domains, but such a domain has not been identified for GPR123 [7]. The second important difference between GPR123 and the other adhesion family members is the absence of a GPCR proteolytic domain (GPS) that functions as an intracellular autocatalytic site [8].

While the precise mechanism for signaling cascades via GPR123 in stem cells is not known, it is generally accepted that adhesion GPCRs use basic alpha subunits of G-proteins, such as Gαs, Gαi, Gαq, and Gα12/13 [4,6].

In the present study, to unravel the role of GPR123 for hPSCs and for hiPSCs generation we suppressed its expression by using RNAi. We demonstrated that the suppression of *GPR123* in hPSCs leads to a loss of pluripotency, alteration in the colony morphology, an accumulation of cells at the G2 phase of the cell cycle, and an absence of the scratch closure in the wound assay, which is associated with decreased cell motility. Downregulation of *GPR123* during the initiation stage of the reprogramming process leads to a decrease in the percentage of the “true” hiPSC colonies, a drop in E-cadherin expression, a decrease in the percentage of NANOG+ nuclei, a loss of GPR123-Gαi co-localization, and the absence of actin cytoskeleton remodeling. This leads to an absence of the alkaline-phosphatase-positive hiPSCs colonies on the 18th day of the reprogramming process. Together, these data indicate for the first time the essential role of GPR123 in pluripotency maintenance as well as pluripotency induction during the reprogramming process.

## 2. Materials and Methods

Cell culture and hiPSCs generation. H9 hESCs (WiCell Research Institute, Madison, MI, USA) and hiPSCs (line SB-NEO1) were cultured in a feeder-free condition on Matrigel-coated plates (Corning Matrigel Matrix, Life Sciences, hESC-qualified, High Wycombe, UK) with mTESR1 media (STEMCELL Technologies, Cambridge, UK). Human iPSC line (SB-NEO1) was generated from the reprogramming of neonatal fibroblasts using the Sendai-based CytoTune-iPS 2.0 Sendai reprogramming kit provided by Life Technologies (A16517, Invitrogen, Fisher Scientific U.K. Ltd.; Loughborough, UK) and described previously [9,10]. Human iPSCs were cultured in the same way as hESCs. The cells were analyzed 24 h, 48 h, 72 h, and 96 h after transfection.

RNA Interference. SMARTpool: siGENOME small interfering RNA (siRNA) for *GPR123* (*ADGRA1*) was purchased from Dharmacon (L-005539-02-0005), siRNA sequences are listed in parentheses (5′-CCACGAACAUCAGGAAUUA-3′, 5′-GGCACACGCUCCUGAAUUU-3′, 5′-GCAGAACGAGCACUCAUUC-3′, 5′-GCACACGGUCAUCCGGUUU-3′). The siRNA mixture at a final concentration of 10 nM was used for transfection with DharmaFECT1 Transfection reagent (Dharmacon, Cambridge, U.K., T-2001-01) according to manufacturer’s instructions with OPTI-MEM reduced serum Media (31985-062; Gibco, Dublin, Ireland) for the first 45 min of transfection. Then, an equal volume of the mTeSR1Medium was added to cells. Media was changed for mTeSR1 every day. As a control, ON-TARGETplus nontargeting control pool from Dharmacon (D-001810-10) was used.

Cell cycle analysis. hESCs and hiPSCs were collected using Accutase (Chemicon, Temecula, CA, USA). Cell cycle analysis was performed using the CycleTest Plus DNA reagent kit (BD Biosciences, Oxford, UK) using a FACS Canto (BD Biosciences) measuring FL2 area versus total counts. The data were analyzed using ModFit LT 4.1. (Verity Software House, Topsham, ME, USA) to generate percentages of cells in G1, S, and G2/M phases. At least 10,000 cells were analyzed in each experiment.

Immunocytochemistry and confocal microscopy. Briefly, hESCs and hiPSCs were cultured on Matrigel-covered glass slide flasks (SlideFlask, NUNC, Roskilde, Denmark) in the mTESR1 media. Cells were quickly washed with phosphate-buffered saline (PBS) prior to being fixed with 2% formaldehyde for 10 min and permeabilized with 0.1% Triton X-100 in PBS for 15 min at room temperature. Unspecific binding was blocked by the incubation of samples in PBS containing 5% normal goat serum for 40 min. Cells were incubated with primary antibodies overnight at 4 °C and with secondary antibodies: goat anti-rabbit-Alexa488 (A-11008, ThermoFisher Scientific, Eugene, OR, USA); goat anti-rabbit-Alexa594 (A-11012, ThermoFisher Scientific, Eugene, OR, USA); goat anti-mouse-Alexa488 (A28175, ThermoFisher Scientific, Bleiswijk, The Netherlands); and goat anti-mouse-Alexa594 (A-11032, ThermoFisher Scientific, Eugene, OR, USA) for 2 h in a dark at room temperature. Primary antibodies used in this study were: anti-GPR123 (PA5-39620, ThermoFisher Scientific, Waltham, MA, USA); anti-GNAI1 (MA5-12800, ThermoFisher Scientific, Bleiswijk, The Netherlands); Rhodamine phalloidin (R415, ThermoFisher Scientific, Eugene, OR, USA); and anti-Nanog (4893 s, Cell Signaling Technology, Danvers, MA, USA). The nuclei were counterstained with DAPI (4′,6-Diamidino-2-Phenylindole, Dihydrochloride; D1306, ThermoFisher Scientific, Eugene, OR, USA). Samples were covered with Vectashield Mounting Medium (Vector Laboratories Ltd., Peterborough, UK). The images were obtained using the Olympus FV3000 (Olympus, Nagano, Japan) microscope and the FluoView FV3000 software (Olympus, Nagano, Japan). At least 100 cells were analyzed for each technical replicate.

For an accurate comparison among immunofluorescence signals of GPR123 (FITC) between emerging hiPSC cells and fibroblasts during the 14th day of the reprogramming process, confocal microscopy images were captured using the same laser excitation and sample emission settings in all immunofluorescence preparations of each slide. In total, 25 fibroblast and 25 hiPSCs from three experimental repeats were analyzed according to Shihan et al. [11]. Negative controls were performed avoiding the primary antibodies. Quantification of the GPR123 immunofluorescence intensity in both cell types was performed with ImageJ software (version 1.53c) [12] and fluorescence values of CTCF (corrected total cell fluorescence) were expressed as arbitrary units/cells according to formula: CTCF = Integrated Density − (Area of selected cell × Mean fluorescence of background readings). The results are represented as the average ± the standard deviations of three independent experiments. We considered *p* values < 0.05 to be statistically significant. *n* = 25 cells for each type.

Transmission electron microscopy. For ultrastructural immunocytochemistry, the cells were fixed in 2.5% glutaraldehyde in 0.1 M cacodylate buffer, pH 7.4, for 1 h at 4 °C, and postfixed in 1% OsO_4_ for 1 h. Then, the cells were mechanically detached from the coverslips and centrifuged. The obtained pellets were dehydrated in graded alcohol solutions and embedded in Epon and Araldit. Prepared with a diamond knife on an LKB-ultratome (Stockholm, Sweden), ultrathin sections were placed on nickel grids, treated with 3% hydrogen peroxide for 20 min to loosen resin, and incubated in the first antibody solution–polyclonal anti-GPR123 (PA5-39620, ThermoFisher Scientific, Waltham, MA, USA) and monoclonal anti- anti-GNAI1 (MA5-12800, ThermoFisher Scientific, MA5-12800 Bleiswijk, The Netherlands) overnight in a moist chamber at 4 °C. After rinsing in PBS containing 0.1% fish gelatin and 0.05% Tween-20, the sections were incubated with secondary anti-mouse and anti-rabbit antibodies conjugated to 10 nm and 15 nm colloidal gold particles, respectively (Sigma, Burlington, ON, Canada). The sections were contrasted with uranyl acetate and lead citrate and examined with a Zeiss Libra 120 electron microscope (Carl Zeiss, Jena, Germany) at an accelerating voltage of 80 kV.

Alkaline-phosphatase staining. AP staining was carried out using the AP Detection kit according to manufacturer’s instructions (Chemicon, Temecula, CA, USA). The images were obtained using the imaging system EVOS FL Auto (ThermoFisher Scientific, Carlsbad, CA, USA) and the EVOS FL Auto 2 Software (ThermoFisher Scientific, Carlsbad, CA, USA).

Flow cytometric analysis for assessing apoptosis. Apoptosis was addressed with the Annexin-V-PE apoptosis detection kit (#556547, BD Bioscience, Oxford, UK) as described previously in [13]. At least 10,000 events were recorded for each sample.

Isolation of RNA and quantitative RT–PCR analysis. To analyze gene expression, total RNA was isolated with Aurum™ Total RNA Mini Kit (BioRad, Hercules, CA, USA) according to the manufacturer’s instructions. RNA was quantified in the NanoDrop ND-1000 Spectrophotometer (NanoDrop Technologies, Inc., Wilmington, DE, USA). cDNA was obtained by reverse transcription of RNA using the RevertAid H Minus First Strand cDNA Synthesis Kit (ThermoFisher Scientific, Vilnius, Lithuania) according to the manufacturer’s instructions. For qRT-PCR, cDNA was amplified with specific primers and the sequences of the oligonucleotides used for the quantitative RT–PCR are shown in Table S1. Regression curves were drawn for each sample and the relative amount was calculated from the threshold cycles with the CFX Manager software for the BioRad CFX-96 real-time system (Bio-Rad, Hercules, CA, USA) based on the manufacturer’s instructions. Relative expression levels of the target genes were normalized with the control gene *GAPDH* or *RPL13A.*

Western immunoblotting. Protein extraction, Western blotting, and antibody/antigen complex detection were performed as published previously [10,14]. Densitometry analysis was performed using ImageLab software Version 6.0.0 (BioRad, Hercules, CA, USA). Glyceraldehyde-3-phosphate dehydrogenase (GAPDH) was used to normalize band intensities of proteins of interest. The details of the antibodies used in this work can be found in the Table S2. The antibody to GAPDH was used after membrane stripping to confirm uniform protein loading.

In brief, cells in 6-well plates were washed with cold-phosphate-buffered saline and lysed in RIPA buffer (50 mM Tris–HCl pH 8.0, 150 mM NaCl, 1% IGEPAL CA 630, 0.5% Na-DOC, and 0.1% SDS). Before the treatment of cells, 1mM PMSF and Roche protease inhibitors (1 tablet per 10 mL) were added to RIPA buffer. After 30 min on ice, the lysates were homogenized and centrifuged at 13,000 r.p.m. for 15 min. The total protein concentration was determined using Pierce™ BCA Protein Assay Kit (#23225, ThermoFisher Scientific, Rockford, IL, USA). Absorption at 595 nm was detected using a Thermo Labsystems Multiskan Ascent. Lysates were electrophoresed on a 8–12% SDS–polyacrylamide gel electrophoresis and electrophoretically transferred to a nitrocellulose membrane (Bio-Rad). Membranes were blocked in Tris-buffered saline with 5% milk and 0.1% Tween. The blots were probed overnight at 4C and the primary antibodies are listed in Table S2. The next morning, the blots were washed and incubated for 2 h with horseradish-peroxidase-conjugated secondary antibodies: Goat Anti-Rabbit HRP (ab205718, Abcam, Cambridge, UK) and Goat Anti-Mouse HRP (ab205719, Abcam, Cambridge, UK). Antibody/antigen complexes were detected using ECL (Amersham Biosciences, Buckinghamshire, UK) and images were acquired using the ChemiDoc MP Imaging System (BioRad, Hercules, CA, USA) and ImageLab 6.0 (BioRad, Hercules, CA, USA) software.

Statistical analysis. All data are demonstrated as mean ± standard deviation (SD) of at least three biological replicates. The statistical tests were performed using GraphPad Prism (Version 7.0) software (GraphPad Software, Boston, MA, USA). Statistical significance between two groups (control RNAi and *GPR123* RNAi) was analyzed using Student’s *t*-test. *p* < 0.05 was considered significant and is denoted as *, *p* < 0.01 as **, and *p* < 0.001 as ***.

## 3. Results

### 3.1. Expression of GPR123 in hESCs and during the Reprogramming Process

**Figure 1:**
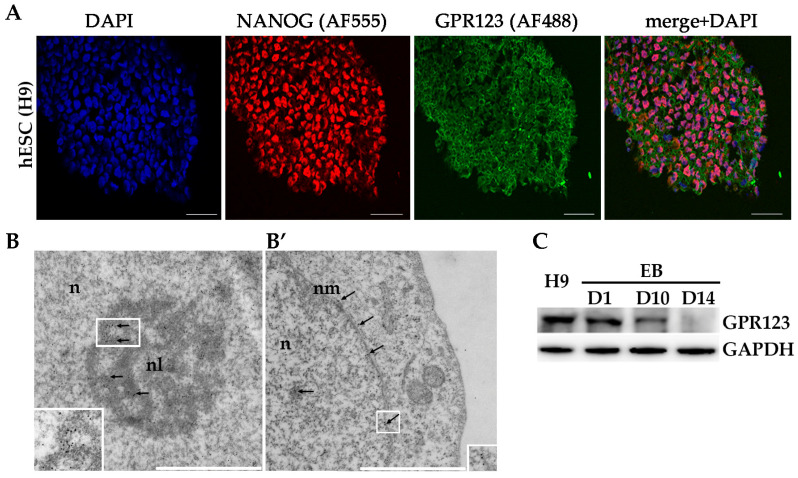

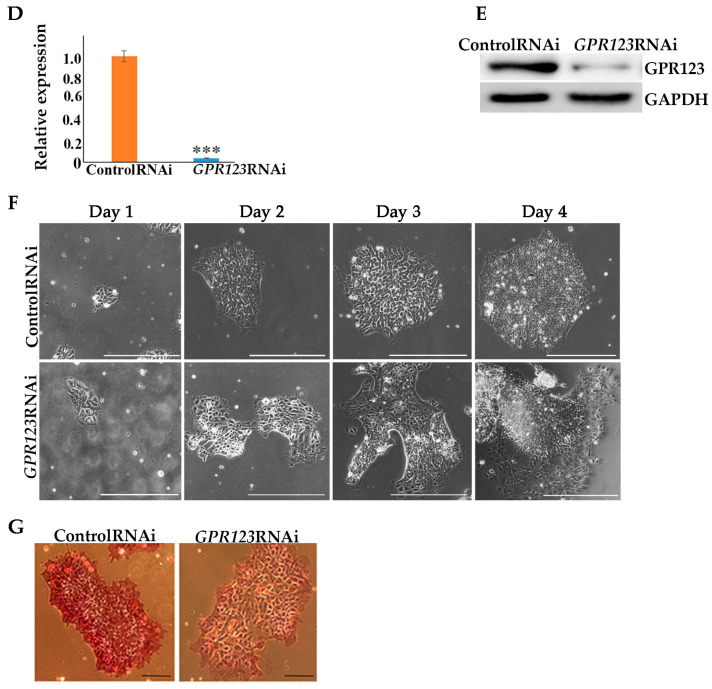
Localization of GPR123 in hESCs and its role in colony morphology. (**A**): Immunofluorescence observation of the GPR122 expression in hESCs. Scale bar 100 μm. (**B**): TEM observation of the GPR123 localization in hESCs. Immunogold labeling (arrows) of the nucleolus (**B**) and nuclear membrane (**B’**) with specific antibody against GPR123 (10 nm gold particles). Abbreviation: *n* stands for nucleus, nl—nucleolus, and nm—nuclear membrane. Inserts at the bottoms (**B**,**B’**) represent GPR123 immunogold labeling at a higher resolution. Scale bar 1 μm. (**C**): Representative Western blot analyses of the GPR123 expression in hESCs (H9) and in embryonic bodies (EB) at day 1 (D1), day 10 (D10), and at day 14 (D14) EBs differentiation. *n* = 3. (**D**): Real-time quantitative polymerase chain reaction analysis of the relative expression of *GPR123* versus *GAPDH* under *GPR123* RNAi in hESCs. Data are shown as mean ± SEM, *n* = 3, with significance difference indicated with asterisks (***, *p* < 0.001). (**E**): Representative Western blot analysis of GPR123 in hESCs transfected with control and *GPR123* RNAi, *n* = 3. GAPDH serve as a loading control. (**F**): Representative images depicting typical colony morphology at phase-contrast observation for the control and *GPR123*RNAi–treated colonies at day 1, day 2, day 3, and day 4 of transfection. Scale bar 400 μm. (**G**): Representative images of the alkaline-phosphatase staining of control and *GPR123* RNAi hESCs (H9). Scale bar 100 μm. Abbreviations: DAPI—4′,6-diamidino-2-phenylindole; hESC—human embryonic stem cell; GAPDH—glyceraldehyde-3-phosphate dehydrogenase; and RNAi—RNA interference.

We first looked at the pattern of GPR123 distribution in the hPSCs, as it was not shown before. Application of the specific antibodies to GPR123 demonstrated that GPR123 is expressed at a high level at the surface of hESCs with residual staining in the cytoplasm (Figure 1A). To reveal GPR123 nuclear localization, we employed transmission electron microscopy (TEM) and observed the accumulation of the numerous immuno-gold particles corresponding to GPR123 at the nucleus, nucleolus, and at the nuclear membrane of the hESCs (Figure 1B,B’). Next, we compared the level of GPR123 expression between hESCs and their differentiated counterparts—embryonic bodies (EBs) (Figure 1C). The obtained results demonstrated that from day 10 of EBs differentiation, the level of GPR123 started to decrease, suggesting that a high level of GPR123 in hESCs indeed could be related to the pluripotent status of cells. To determine what role GPR123 plays in hPSCs, we achieved a high level of *GPR123* gene suppression by RNAi (Figure 1D,E) and observed significant changes in colony morphology as early as day 2 from transfection experiments (Figure 1F). Namely, in addition to pronounced changes in the shape of the colonies, the cells in the colonies significantly increased in size. By the fourth day, the colonies began to grow upwards into a dome-shaped form (Figure 1F) and were characterized by the absence, or very weak staining, of alkaline phosphatase (AP) in contrast to control RNAi cells (Figure 1G).

It is well accepted that maintenance of the pluripotency is tightly regulated and reflected by the characteristic hPSC colony morphology. Thus, the revealed morphological changes in the colonies indicated the important role of GPR123, not only for pluripotency maintenance in established clones, but it also suggests its role for pluripotency acquisition during the reprogramming process. To further understand the involvement of GPR123 in pluripotency acquisition, we employed *GPR123* RNAi at the same time as we performed the screen of the Dharmacon library, i.e., from the 8th to the 10th day of the reprogramming process [1].

**Figure 2:**
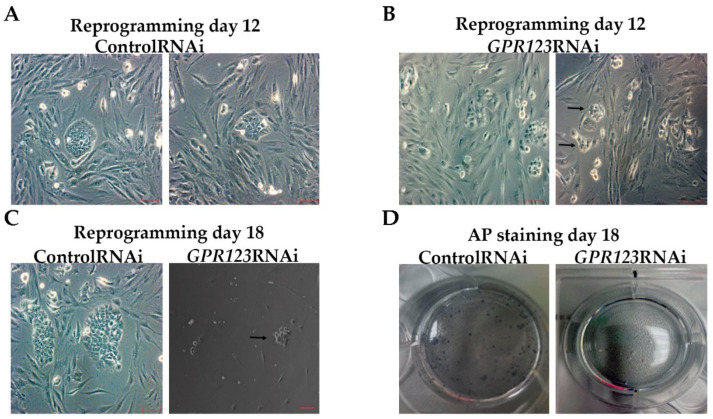

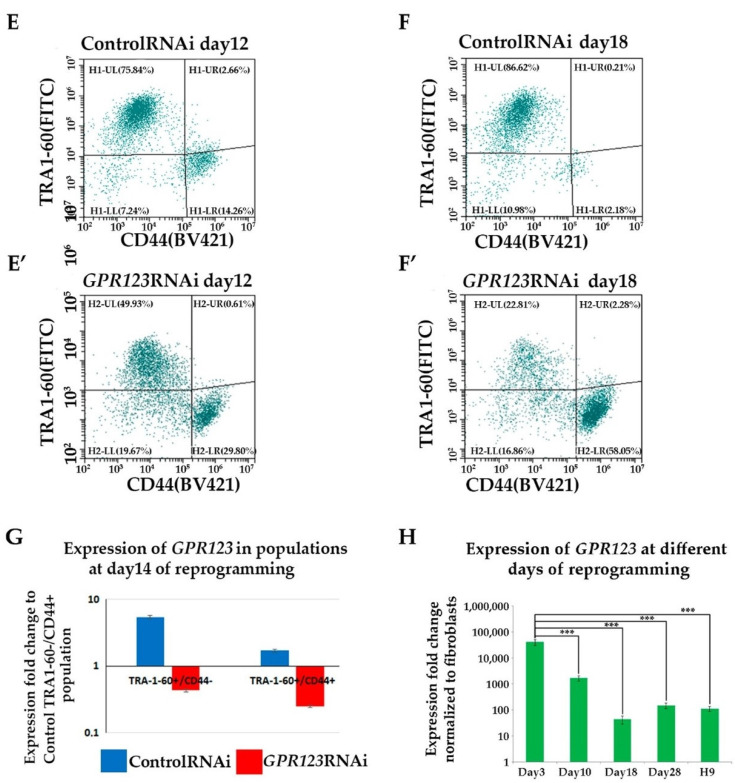
Downregulation of GPR123 abrogates human-induced pluripotent stem cells generation. (**A**): Phase-contrast observation of the typical hiPSC colonies at day 12 of the reprogramming process under the control RNAi (**A**) and *GPR123* RNAi (**B**). Arrows poined to hiPSCs colonies. Scale bar 100 μm. (**C**): Representative image of the typical colonies at day 18 of the reprogramming process under the control RNAi and *GPR123* RNAi. Arrows poined to hiPSCs colonies. Scale bar 100 μm. (**D**): Representative images of the alkaline-phosphatase (AP) staining of the control and *GPR123* RNAi hiPSCs at day 18 of reprogramming. (**E**,**E’**,**F**,**F’**): Flow cytometry analysis of different subpopulations during the time course (at day 12 and day 18) of reprogramming in the control and *GPR123* RNAi-treated groups. (**G**): Real-time quantitative polymerase chain reaction analysis of the relative expression of GPR123 in TRA-1-60+/CD44- populations (true iPSCs) and in partly reprogrammed cells (TRA-1-60+/CD44+) at day 14 in the control and *GPR123* RNAi groups. Data are shown as mean ± SEM, *n* = 3. (H): Real-time quantitative polymerase chain reaction analysis of the *GPR123* expression normalized to expression at the neo1 fibroblasts during the time-course of transduction. Data are shown as mean ± SEM, *n* = 3, with the significance difference indicated with asterisks (***, *p* < 0.001). Abbreviations: AP—alkaline phosphatase.

In agreement with the data obtained by us previously by high-throughput small interfering RNA (RNAi) screening assay, which allowed specific knockdown of the 784 members of the different kinases and phosphatases from the Dharmacon library during the initiation phase of reprogramming [1], we observed significant downregulation in the number of the hiPSCs colonies from day 12 to day 18 of the reprogramming period (Figure 2A–C) with complete absence of the AF+ colonies at day 18 in the *GPR123* RNAi group (Figure 2D). Immunoflow examination of the different populations during the reprogramming under *GPR123* RNAi demonstrated a decrease in the percentage of the TRA-1-60+/CD44- population in the *GPR123* RNAi group by day 18 down to 22.8% compared with 86.6% in the control RNAi group (Figure 2E,E’,F,F’), emphasizing the role of this gene in the generation of hiPSCs. Further comparison of the *GPR123* gene expression at day 14 in the TRA160+/CD44-sorted population, corresponding to the “true” hiPSCs and in TRA1-60+/CD44+ sorted cells, represented by the population at the intermediate state of reprogramming, demonstrated that compared with the control RNAi cells, the expression of the *GPR123* gene drops in both populations of the *GPR123* RNAi cells (Figure 2G). Next, to determine how the level of the *GPR123* expression changes during the entire reprogramming process, we checked its level at all stages. The analysis showed that the highest level of the *GPR123* is observed at the initiation stage. Further, the expression level gradually decreased and by the end of the reprogramming process, on day 28, it practically did not differ from the level in the H9 cells (Figure 2H).

Thus, our findings indicate that elevation of *GPR123* expression during the early stages of the reprogramming process is important and a necessary requirement for induction and maintaining pluripotency.

### 3.2. Downregulation of GPR123 Expression Causes Loss of Pluripotency Leading to hESCs Differentiation and Abrogation of hiPSCs Colonies during the Process of Reprogramming

**Figure 3:**
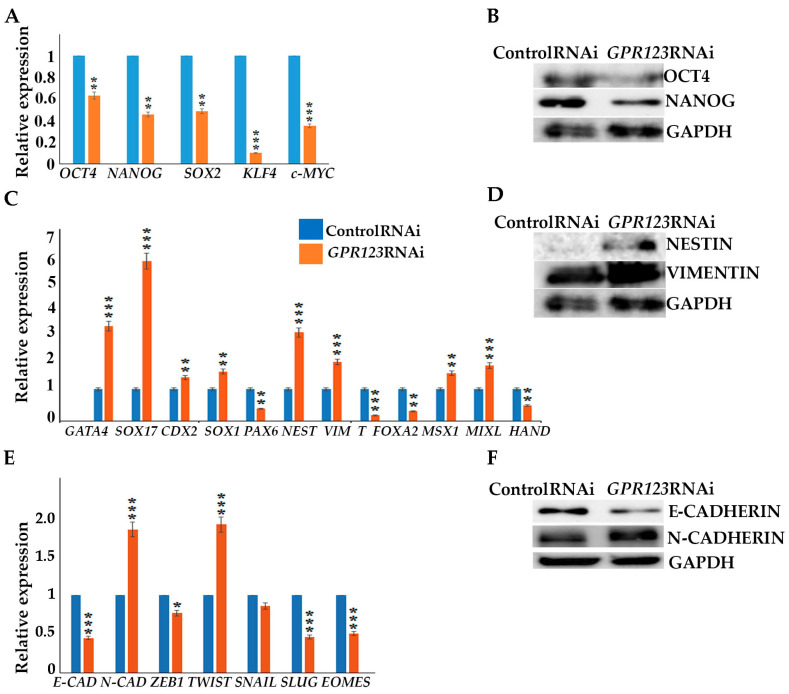

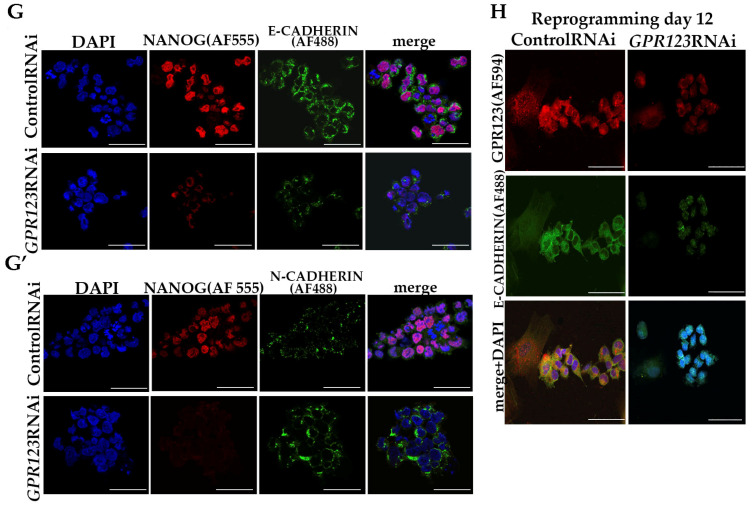
Downregulation of GPR123 results in loss of pluripotency and increased expression of differentiation marker genes in hPSCs. (**A**): Real-time quantitative PCR analysis of *OCT4*, *NANOG*, *SOX2*, *KLF4*, and *c-MYC* expression in hESCs (H9) control and *GPR123* RNAi groups. Data represent relative expression to *GAPDH* and were normalized against the control RNAi. Results are presented as mean ± SEM (*n* = 3), with significance difference indicated with asterisks (*p* < 0.01 as **, *p* < 0.001 as ***). (**B**): Representative Western blot analyses of the pluripotent markers OCT4 and NANOG expression in hESCs (H9) treated with the control and *GPR123* RNAi. (**C**): Real-time quantitative PCR analysis of the differentiation markers expression in hESCs (H9) control and *GPR123* RNAi groups. Data represent relative expression to *GAPDH* and were normalized against the control RNAi. Results are presented as mean ± SEM (*n* = 3), with significance difference indicated with asterisks (*p* < 0.01 as **, *p* < 0.001 as ***). (**D**): Representative Western blot analyses of the differentiation markers NESTIN and VIMENTIN expression in hESCs (H9) treated with the control and *GPR123* RNAi. (**E**): Real-time quantitative PCR analysis of MET genes expression in hESCs (H9) control and *GPR123* RNAi groups. Data represent relative expression to *GAPDH* and were normalized against the control RNAi. Results are presented as mean ±SEM (*n* = 3), with significance difference indicated with asterisks (*p* < 0.05 as *, *p* < 0.001 as ***). (**F**): Representative Western blot analyses of the E-cadherin and N-cadherin expression in hESCs (H9) treated with the control and *GPR123* RNAi. (**G**,**G’**): Confocal immunofluorescence observation of the NANOG and E-cadherin expression in the control and *GPR123* RNAi hESCs (H9). Scale bar 50 μm. (**H**): Representative confocal immunofluorescence images of E-cadherin expression in the control and *GPR123* RNAi hiPSCs at day 12 of the reprogramming. Scale bar 50 μm.

Serious changes identified in the morphology of the colonies under *GPR123* RNAi suggested loss of pluripotent characteristics and induction of the differentiation process. Thereby, we examined expression levels of the core pluripotent markers and observed significant downregulation of *OCT4, NANOG, SOX2, KLF4*, and *c-Myc* (Figure 3A,B) with a simultaneous increase in expression of the three layers of germ cell markers, namely, *GATA4, SOX17, NESTIN, Vimentin, MSX1*, and *MIXL* (Figure 3C,D). In addition, we paid special attention to genes, whose expression is very important for a successful hiPSCs generation, especially during mesenchymal–ephitelia transition (MET). During MET, downregulation of N-cadherin expression and induction of E-cadherin are required for successful iPSCs generation [1,9,10]. However, we found that in hESCs, *GPR123* RNAi causes a drop in E-cadherin expression but a significant rise in N-cadherin and twist levels (Figure 3E,F). Immunofluorescence examination confirmed the downregulation of E-cadherin and upregulation of N-cadherin alongside the loss of NANOG expression in *GPR123* RNAi hESCs colonies (Figure 3G,G’). In accordance with these, we observed a very weak pattern of E-cadherin staining in induced colonies at Day 12 of the reprogramming process under *GPR123* RNAi, while a strong pattern of E-cadherin was found in the control RNAi colonies (Figure 3H). Moreover, confocal immunofluorescence observation revealed co-localization of E-cadherin and GPR123 at the surface of the control RNAi cells (Figure 3H).

Since downregulation of *GPR123* in hESCs leads to a significant decrease in the expression level of pluripotent markers, including NANOG, the expression of which precedes the expression of OCT4 during the reprogramming process, we examined NANOG expression in the emerging colonies on days 12 and 18 of the reprogramming process. Our analyses demonstrated that on day 18 of reprogramming, 64% of the nuclei in the control RNAi group were positive for NANOG, while only 4% of the positively stained nuclei were found in the *GPR123* RNAi group (Figure S1A,B), pointing to an important role of GPR123 for NANOG expression.

### 3.3. GPR123 Suppression Leads to the Accumulation of hESCs at the G2 Phase of the Cell Cycle, but Does Not Cause Apoptosis

**Figure 4:**
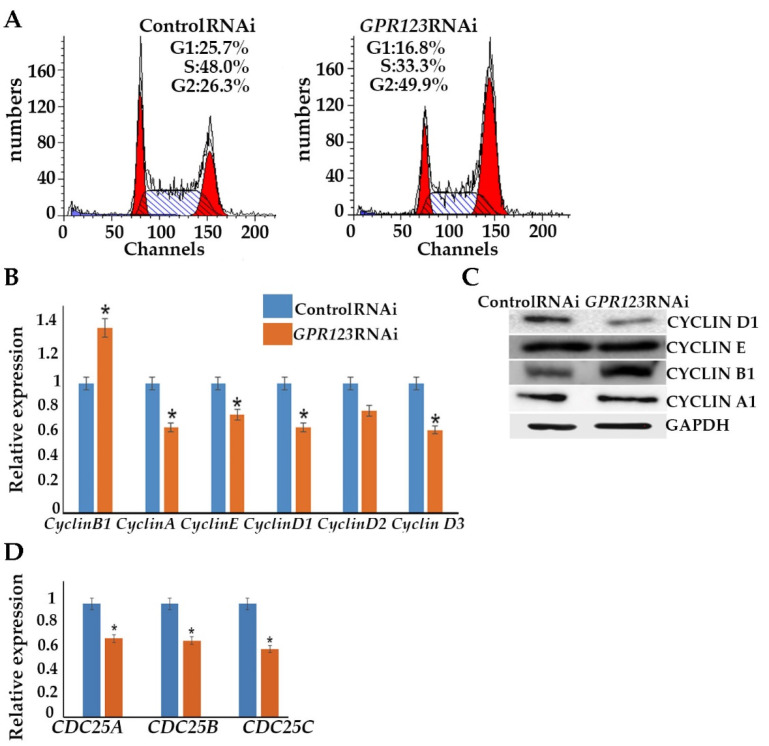

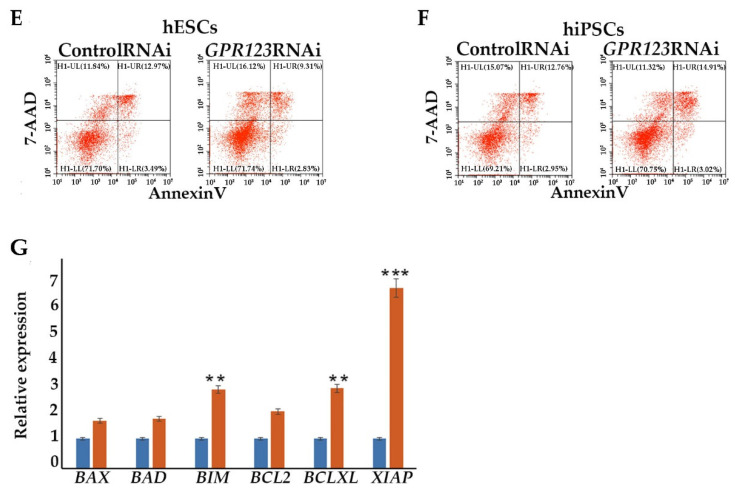
Downregulation of GPR123 leads to accumulation of cells at the G2 phase of the cell cycle. (**A**): MODFIT analysis of the cell cycle of hESCs (H9) treated with the control and *GPR123* RNAi. (**B**): Real-time quantitative PCR analysis of the cell cycle genes expression in hESCs (H9) control and *GPR123* RNAi groups. Data represent relative expression to *GAPDH* and were normalized against the control RNAi. Results are presented as mean–SEM (*n* = 3), with significance difference indicated with asterisks (*p* < 0.05 as *). (**C**): Representative Western blot analyses of the CYCLIN D1, CYCLIN E, CYCLIN B1, and CYCLINA1 in control and *GPR123* RNAi hESCs (H9). (**D**): Real-time quantitative PCR analysis of the *CDC25A*, *CDC25B,* and *CDC25C* genes expression in hESCs (H9) control and *GPR123* RNAi groups. Data represent relative expression to *GAPDH* and were normalized against the control RNAi. Results are presented as mean –SEM (*n* = 3), with significance difference indicated with asterisks (*p* < 0.05 as *). (**E**,**F**): Flow cytometric analysis of apoptosis in hESCs (H9). (**E**,**F**): The hiPSCs under treatment with Control and *GPR123* RNAi. (**G**): Real-time quantitative PCR analysis of the apoptosis genes expression in hESCs (H9) control and *GPR123* RNAi groups. Data represent relative expression to *GAPDH* and were normalized against the control RNAi. Results are presented as mean –SEM (*n* = 3), with significance difference indicated with asterisks (*p* < 0.01 as **, *p* < 0.001 as ***).

A change in the cell proliferation profile from the one that is generally attributed to somatic cells to a faster and shorter hESC-like cycle is a prerequisite of successful iPSC generation [15]. We employed flow cytometry to examine the cell cycle profile of the control and *GPR123* RNAi hESCs (Figure 4A). In agreement with the discovered downregulation of the pluripotency markers’ gene expression, we observed that on the 3rd day of transfection, about 50% of the *GPR123* RNAi cells demonstrated accumulation at the G2 phase of the cell cycle in contrast to control RNAi cells (Figure 4A). This was further supported by the drop in expression of the D-type cyclins and cyclin E and by the rise of the important regulator of the G2 phase progression, cyclinB1 (Figure 4B,C). Observed downregulation in the expression level of all three main phosphatases, namely, CDC25A, CDC25B, and CDC25C, which governed cell cycle progression from one stage to another, suggests that the cell cycle slows down (Figure 4D). Previously, we showed that cell cycle alteration accompanied by the downregulation of the pluripotency gene expression and accumulation of hESCs at the G2 phase of the cell cycle may not lead to increased cell death and apoptosis induction [16]. We used flow cytometry to examine the level of 7-AAD and AnnexinV in *GPR123-RNAi-treated* hESCs and hiPSCs (Figure 4E,F) and as expected found no significant difference in the rate of apoptosis between the control RNAi and *GPR123* RNAi groups in both cell types. Further qRT-PCR revealed upregulation of the pro-apoptotic BCL2-family gene expression, namely, the BIM gene, in the *GPR123* RNAi group with contaminant upregulation of Bcl-xL, which functions to inhibit apoptosis by a number of different mechanisms including inhibition of Bax. In addition, the X-linked inhibitor of apoptosis protein (XIAP), the most potent and best-defined anti-apoptotic IAP family member, appears to be significantly upregulated in *GPR123* RNAi cells, suggesting that the fine balance between pro-and anti-apoptotic genes was maintained during the suppression of *GPR123* gene expression (Figure 4G). These data are consistent with the idea that hPSCs prefer a differentiation pathway to apoptosis induction for eliminating cells with a reduced level of pluripotency [17].

### 3.4. Gαi Is an Important Partner for Signal Transduction by GPR123 in Pluripotent Stem Cells

**Figure 5:**
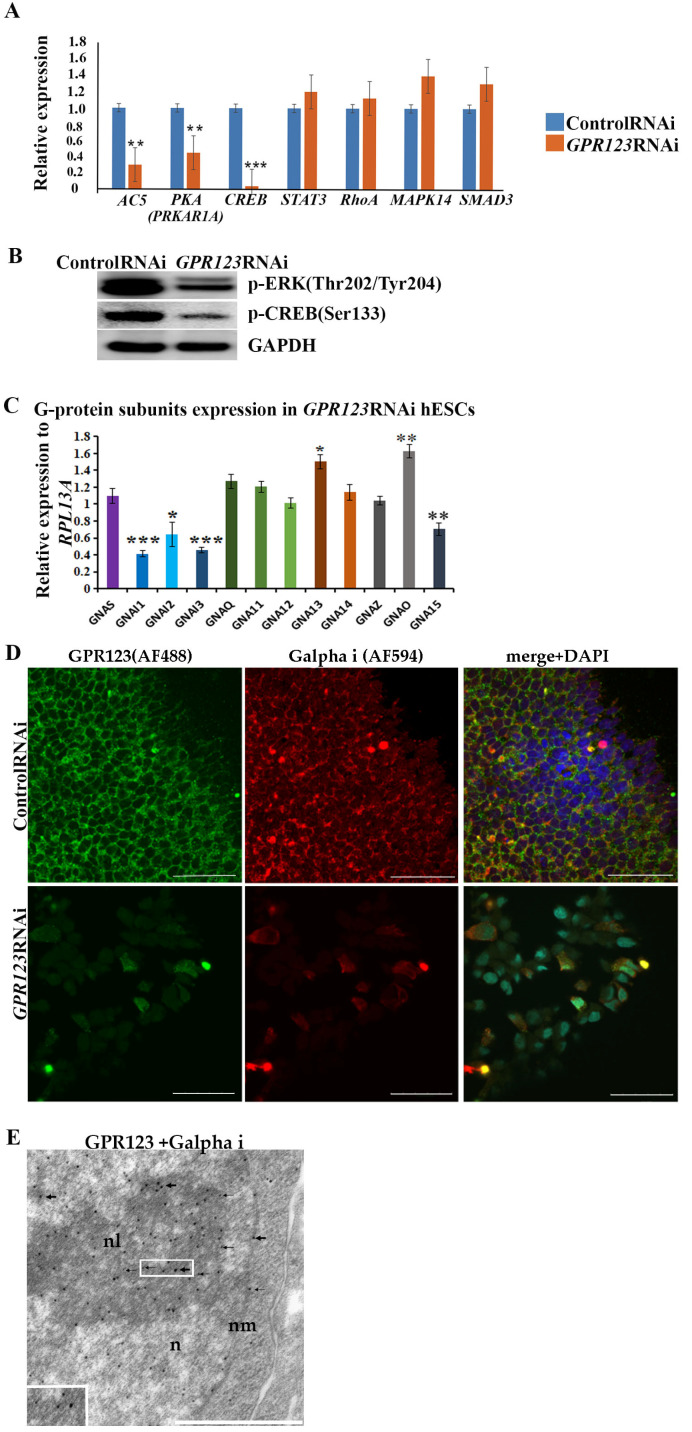
Expression and co-localization of Gαi and GPR123 in hESCs. (**A**): Real-time quantitative PCR analysis of the *AC5*, *PKA, CREB*, *STAT3*, *RhoA*, *MAPK14*, and *SMAD3* genes expression in hESCs (H9) control and *GPR123* RNAi groups. Data represent relative expression to *GAPDH* and were normalized against the control RNAi. Results are presented as mean –SEM (*n* = 3), with significance difference indicated with asterisks (*p* < 0.01 as **, *p* < 0.001 as ***). (**B**): Representative Western blot analyses of the p-ERK (Thr202/Tyr204) and p-CREB (Ser133) in the control and *GPR123* RNAi hESCs (H9). (**C**): Real-time quantitative PCR analysis of the Gα subunits expression in *GPR123* RNAi hESCs (H9). Data represent relative expression to *RPL13A* and were normalized against expression of the Gα subunits in the control RNAi hESC line (H9). Results are presented as mean ± SEM (*n* = 3), statistical significance was analyzed using Student’s *t*-test, *p* < 0.05 was considered significant and is denoted as *, *p* < 0.01 as **, *p* < 0.001 as ***. (**D**): Representative images of the Confocal immunofluorescence observation of the co-localization between Gαi with GPR123 in hESCs (H9) treated with the control RNAi (upper panel) and with *GPR123* RNAi (bottom panel). Scale bar 50 μm. (**E**): TEM observation of the Gαi and GPR123 localization in hESCs. Immunogold labeling in the nucleus (*n*), nuclear membrane (nm), and in the nucleolus (nL) with specific antibodies against the Gαi (15 nm gold particles, black thick arrows) and GPR123 (10 nm gold particles, black thin arrows). An inset in the lower left corner depicts the area with both labels. Scale bar 1 μm.

Morphological changes observed in the *GPR123* RNAi hESCs/hiPSCs colonies, especially accompanied by colonies’ retraction inwards (Figure 1F), suggest that GαS and Gαi might be involved in this process [5,18]. We assumed that the signaling pathway of adenylate cyclase (AC5) -cAMP-PKA-CREB for GαS subunit and cAMP-ERK1/2–CREB-for Gαi signaling would be among the main streams from GPR123 [6,18]. Indeed, qRT-PCR data support a significant decrease in the level of AC-PKA and CREB expression in *GPR123* RNAi cells vs. control RNAi cells (Figure 5A). At the same time, qRT-PCR analysis of other downstream targets such as for Gαq/11 (*STAT3*) and Gα 12/13 (*MAPK14*) did not reveal significant alteration in their expression levels (Figure 5A,B).

In mouse ESCs, cholera toxin permanently activate GαS leading to cAMP generation and further phosphorylation of CREB, suggesting that the GαS-cAMP cascade contributes to pluripotency maintenance [18]. At the same time, suppression of Gαi by the pertussis toxin leads to significant changes in the hESC colony morphology, a decrease in the level of alkaline-phosphatase-positive colonies, and invagination of colonies [5], thus supporting our assumption of the involvement of the Gαi signaling in the observed morphological changes of hPSC colonies under *GPR123* RNAi. To clarify this, we evaluated the expression level of the main G alpha subunits in hESCs treated with *GPR123* RNAi by qRT-PCR. It appears that expression levels of the three G alpha i subunits, namely, *GNAI1* (G Protein Subunit Alpha I1), *GNAI2* (G Protein Subunit Alpha I2), and G protein subunit alpha i3 (*GNAI3*) involved in the regulation of cAMP and CREB pathways were significantly downregulated (Figure 5C).

Next to this, we employed the confocal immunofluorescence analysis of the Gαi staining pattern in the control and *GPR123* RNAi hESCs, which revealed co-localization between GPR123 and the Gαi subunit in the control group and a very weak to no expression and co-localization between GPR123 and Gαi in *GPR123* RNAi hESCs (Figure 5D), corroborating the qRT-PCR data. In addition, transmission electron microscopy (TEM) demonstrated close localization of the immunogold particles corresponding to GPR123 and Gαi not only in the cytoplasm, but also in the perinucleolar space, on the nuclear membranes and in the chromatin (Figure 5E). Moreover, immunofluorescent analysis performed on the 12th day of reprogramming showed the co-localization between Gαi and GPR123 in emerging hiPSCs, similar to the one observed earlier in hESCs (Figure 5D and Figure S2A). Suppression of *GPR123* expression by RNAi leads to a decrease in Gαi expression both in hESCs and in hiPSCs colonies and, accordingly, to the loss of such co-localization (Figure 5D and Figure S2B). Thus, we concluded that in hPSCs, the Gαi represents an important partner of GPR123.

Importantly, we noticed that in hiPSCs colonies that arose during the reprogramming process, the pattern of the immunofluorescence staying level for GPR123 is much higher than in surrounding fibroblasts (Figure S2A). To verify this observation, we employed the ImageJ 1.53t (National Institutes of Health, Bethesda, MD, USA) software tool to measure the immunofluorescent intensity of GPR123 in both cell types. Indeed, the obtained result confirmed that the immunofluorescence level of the GPR123 is much higher in emerging hiPSC cells (Figure S2B), thus further supporting our qRT-PCR data on sorted populations during the reprogramming process about a high level of *GPR123* expression in TRA1-60+/CD44- cells in contrast to TRA1-60-/CD44+ cell population, which was refractory to reprogramming (Figure 2G).

### 3.5. Downregulation of GPR123 Leads to the Loss of Cellular Motility

**Figure 6:**
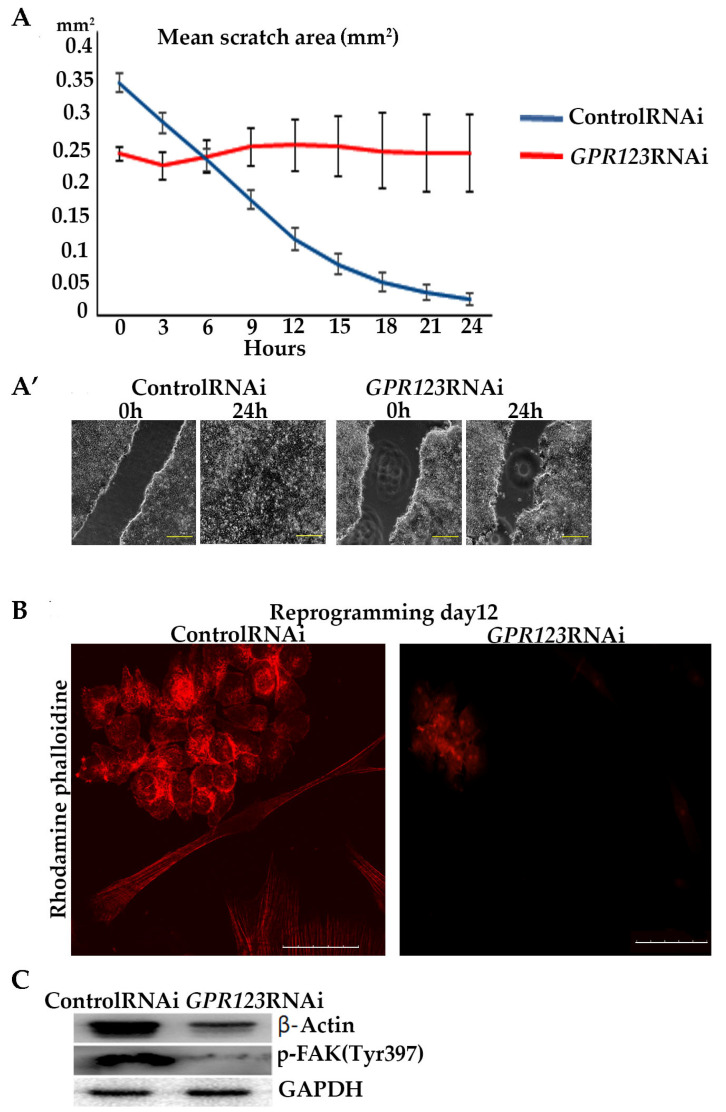
GPR123 is important for wound healing of hiPSCs and ACTIN reorganization during the reprogramming process. (**A**): Graphical representation of the wound healing of hiPSCs treated with the control and *GPR123* RNAi. Human iPSCs were subjected to scratch wounding from 0 h until 24 h (*n* = 5). (**A’**): Representative images of the time-lapse phase-contrast observation of the wound healing at time 0 and 24hrs. Scale bar 200 µm. (**B**): Representative images of the confocal immunofluorescent staining with Rhodamine phalloidine in the control and *GPR123* RNAi hiPSCs. Scale bar 50 μm. (**C**): Representative Western blot analyses of β-ACTIN and p-FAK(Tyr 397) in hiPSCs treated with the control and *GPR123* RNAi.

We hypothesized that impaired co-localization between Gαi and GPR123 in *GPR123* RNAi colonies will result in disturbances of the signaling cascades important for cellular motility. The importance of the Gαi subunit for hPSC colony morphology and motility was demonstrated before by experiments with the pertussis toxin, which suppresses Gαi signaling, causing the absence of overgrowth of the scratch in the hPSCs [5]. We performed scratch overgrowth experiments in the control RNAi and in *GPR123* RNAi hiPSCs. As expected, the obtained results demonstrated the absence of scratch closure in *GPR123* RNAi colonies (Figure 6A,A’), thus further suggesting impaired Gαi-GPR123 signaling in our experimental condition. These results are consistent with the observed lack of actin cytoskeleton remodeling, a prerequisite for the successful formation of induced pluripotent stem cell colonies during the reprogramming process, decreased expression of β-Actin, and focal adhesion (p-FAKTyr397) in *GPR123* RNAi colonies (Figure 6B,C) [1], indicating that the GPR126-Gαi signaling axis is important and participates in the regulation of hPSC movement.

## 4. Discussion

Stem cell technology is mainly dedicated to practical applications for regenerative medicine, disease modeling, drug screening, and understanding of human developmental biology. Currently, successful clinical trials with the use of stem cells are being carried out, as well as studies using stem cells in disease modeling related to the musculoskeletal system, heart, nervous system, immune system, etc. [19,20]. However, with such a widespread application of hiPSCs, our knowledge and understanding of the entire mechanism of the reprogramming process is still very limited. At the same time, successful clinical applications of hiPSCs will require overcoming serious downsides, one of which is incomplete reprogramming, which calls for a deeper understanding of the molecular machinery of the reprogramming process. Therefore, our new data highlighting the importance of the GPR123 and GPR123-Gαi signaling in hiPSCs generation could be the first step in this direction.

As mentioned here, GPR123 is an orphan receptor. Data on its function even in somatic cells are very limited [4]. Further research is needed to discover specific agonists or antagonists for adhesion GPCRs, including GPR123. For example, recently, beclomethasone dipropionate was identified as a small molecular weight agonistic compound for GPR97 [21,22].

Many GPCRs have been shown to bind to more than one member of the G protein family. Adhesion GPCRs are no exception to this rule. For example, GPR126 and GPR133 interact with both Gαs and Gαi proteins [23]. GPR64 interacts with Gαs and Gαq proteins [24] and GPR56 binds to Gαq/11 and Gα12/13 [25].

It is still largely a mystery how autoproteolysis and NTF removal occur. The signaling pathways associated with the above events may differ for different receptors and even for the same receptor depending on the cellular context. The structural features of the GPR123 molecule suggest that not all adhesion GPCRs rely on released NTF for their signaling [21,26]. This is supported by studies of lat-1 in C. elegans showing that separation of NTF and CTF is not necessary to achieve proper receptor function [27]. For most adhesive GPCRs, their large size is determined by the NTF domain, comprising modular protein domains, such as cadherin, epidermal growth factor, immunoglobulin, and leucine-rich repeat domains. Some of these domains may mediate contacts with other cells or extracellular matrix-associated molecules. However, as noted earlier here, most of the adhesion GPCRs remain orphaned in terms of ligand binding [4,19]. In this context, more work is required to explain our findings on the co-localization of GPR123 and E-cadherin in hPSCs. Currently, there is no doubt that various GPCRs play an important role in the maintenance of stem cells and in reprogramming towards hiPSCs [1,5,6,18]. Moreover, serious changes in the gene expression of some GPCRs at different stages of stem cell differentiation once again emphasize their involvement in the maintenance of stemness [28]. Our data showing that during the course of EBs differentiation the protein level of GPR123 demonstrated significant reduction are in good agreement with data about a significant drop at GPR123 during specification to ectoderm differentiation of the HUES64 hESC line. Throughout comprehensive transcriptional profiling of populations derived through directed differentiation of hESCs, Gifford and colleagues identified GPR123 among the most downregulated GPCRS, highlighting that a better understanding of the involvement of GPCRs in the specification events can lead to the development of the more effective differentiation strategies. Analysis of gene expression profiles performed by Choi and colleagues demonstrated that more than 106 GPCRs were over expressed in the PCSs or cancer stem cells, whereas the expression of the other 22 GPCRs was downregulated and 81 were differentially expressed during somatic reprogramming to iPSCs [6]. However, since that time, very little has been done to shed light on the role and function of these molecules for pluripotency maintenance and other biological properties of hPSCs.

Here, we have shown that the adhesion family member GPCR, GPR123, is essential for pluripotency maintenance of hPSCs as well as for hiPSCs generation:

(i). By employing *GPR123* RNAi from the 8th to the 10th day of the reprogramming process, we demonstrated significant downregulation in the number of hiPSCs colonies from the 12th to the 18th day of the reprogramming period, with complete absence of the AP+ colonies on the 18th day.
(ii). Flowcytometry cell populations analysis demonstrated significant decrease in the percentage of the “true” hiPSCs (TRA1-60+/CD44- population) by the 18th day of the reprogramming process under *GPR123* RNAi.
(iii). We demonstrated significantly reduced expression of *GPR123* in the emerging TRA1-60+/CD44- population and in the population of the partially reprogrammed cells (TRA+CD44+) from the *GPR123* RNAi group on the 14th day of the reprogramming process.

Thus, our findings indicate that elevation of *GPR123* expression during the initial stages of the reprogramming process is an important and necessary requirement for induction and maintaining pluripotency during this process, corroborating previous data that the expression level of GPR123 in hESCs is much higher than in 100 somatic cell types tested by Nakamura and colleagues [5].

We addressed the effect of *GPR123* suppression on pluripotency maintenance as one of the most important characteristics of hPSCs and demonstrated an important role of GPR123 in pluripotency maintenance as:

(iv). Suppression of *GPR123* by RNAi leads to significant downregulation of *OCT4*, *NANOG*, *SOX2*, *KLF4*, and *c-Myc* pluripotency markers expression
(v). with simultaneous increase in expression of differentiation markers genes, namely *GATA4*, *SOX17*, *NESTIN*, *Vimentin*, *MSX1* and *MIXL*.
(vi). *GPR123* RNAi causes a drop in E-cadherin expression with a significant rise in N-cadherin.
(vii). Confocal immunofluorescence observation revealed co-localization of E-cadherin and GPR123 at the surface of the control RNAi cells and the absence of such colocalization in the *GPR123* RNAi population.

The close relationship between pluripotency and cell cycle regulation is well documented [14,16,29,30] and alterations in the expression of the cyclins and CDKs lead to abrogation of the hiPSCs [1,9,10]. For this reason, we examine the cell cycle profile of the control and *GPR123* RNAi hESCs and observed accumulation of the *GPR123* RNAi cells at the G2 phase of the cell cycle of the third day of transfection. This was further supported by the increased level of the cyclinB1 level. As was previously mentioned, one of the important prerequisites of successful hiPSCs generation is the acceleration of the cell cycle [15]. Thus, accumulation of the *GPR123* RNAi cells at the G2 phase of the cell cycle might be one of the reasons for the absence of the hiPSCs colonies under *GPR123* RNAi. Therefore, we concluded that:

(viii). Expression of GPR123 is important for proper cell cycle regulation in hPSCs.

GPCRs are the largest family of cell surface receptors that modulate the activity of a variety of intracellular signals via G-protein signaling. G proteins are second messengers in intracellular signaling and consist of Gα, Gβ, and Gγ subunits. Gα subunits are subdivided into four subfamilies according to their structural and functional features: Gαs, Gαi/o, Gαq/11, and Gα12/13 [31]. The G alpha (α) subunits partners are not known for GPR123, but it is expected that adhesion GPCRs can transduce signals via main G alpha subunits, including GαS and Gαi [6,21]. Previously, it was shown that hPSC colonies form and maintain characteristic pluripotent morphology and organization through Gαi function [5]. Observed morphological changes in colonies under *GPR123* RNAi allow us to suggest that Gαi subunits may be involved in this process. To determine which G α subunits might be affected by *GPR123* RNAi in hPSCs, we analyzed the expression pattern of the main G α subunits by qRT-PCR and revealed that:

(ix). Expression levels of all three G alpha i subunits: *GNAI1*, *GNAI2*, and *GNAI3* involved in regulation of cAMP and CREB pathways were significantly downregulated.

Corroborating the qRT-PCR data, confocal immunofluorescence analysis of the Gαi-staining pattern in the control and *GPR123* RNAi hPSCs revealed a co-localization between GPR123 and the Gαi subunit in the control cells and loss of such co-localization between GPR123 and Gαi in *GPR123* RNAi cells. Importantly, similar observation confirms loss of such co-localization on the 12th day of reprogramming in emerging hiPSC colonies.

Based on the above data, we concluded that in hPSCs the Gαi represents an important partner for GPR123 and the GPR123–Gαi axis is important for hPSC pluripotency maintenance and acquisition.

In addition to carrying out signaling cascades associated with the activation of cAMP and CREB, Gαi is also actively involved in cellular processes such as cell adhesion, cytoskeleton remodeling, actin nucleation and, accordingly, associated with cell mobility [32,33]. To reveal involvement of the GPR123–Gαi axis in cell motility, we performed scratch-overgrowth experiments in control RNAi and in *GPR123* RNAi hiPSCs. Our data demonstrated the absence of scratch closure in *GPR123* RNAi colonies in good agreement with the previous data of Nakamura and colleagues [5]. In their work by experiments with the pertussis toxin, which suppresses Gαi signaling, these authors demonstrated the absence of overgrowth of the scratch in hPSCs. Having in mind a reduced expression of all three Gαi subunits in *GPR123* RNAi cells, we assumed that in our experimental settings, signaling cascades, which are important for cell motility and regulated by the GPR123–Gαi axis, are impaired. Thus, the GPR123–Gαi axis is important for cell motility in hPSCs.

Summarizing the obtained results, we can conclude that a high level of GPR123 expression is important for both: (1) the maintenance of pluripotency in hPSCs and (2) its acquisition during the reprogramming. All the above data are fundamentally new and deserve further attention.

Given the evidence discussed herein, the significance of the GPR123 in stem cell maintenance and somatic reprogramming to hiPSCs allows us to consider GPR123 as a new important hPSC marker, thus highlighting the need for further extensive research on its regulation. Our present and previous data [1] demonstrate that GPCRs are a promising target for modulating the formation and organization of hPSC colonies and are important for understanding somatic cell reprogramming.

## 5. Conclusions

The results described herein allow us to conclude that GPR123 performs essential functions in hESCs and is necessary for pluripotency maintenance in hPSCs as well as for its acquisition during the reprogramming. Specifically, the suppression of *GPR123* expression by RNAi leads to the loss of pluripotency, differentiation, and accumulation of cells at the G2 phase of the cell cycle. Application of the *GPR123* RNAi from days 8 to 10 of reprogramming leads to a decrease in the percentage of the “true” hiPSC colonies, a drop in E-cadherin expression, a decrease in the percentage of NANOG+ nuclei, reduced cell motility, and the absence of actin cytoskeleton remodeling. Together, these lead to the absence of hiPSCs colonies on the 18th day of the reprogramming process. Therefore, this study identifies GPR123 as an important pluripotency-associated effector, providing new insight into the interplay between adhesion GPR123 and pluripotent reprogramming.

## Data Availability

Not applicable.

## Supplementary Materials

The following supporting information can be downloaded at: https://www.mdpi.com/article/10.3390/cells12020304/s1, Figure S1: Downregulation of GPR123 significantly reduced the percentage of NANOG positive cells during the reprogramming process; Figure S2: Co-localization between Gαi and GPR123 is lost under *GPR123* RNAi in hiPSCs at day 12 of the reprogramming process; Table S1: List of the primers for qRT-PCR used in the present study; Table S2: List of the primary antibodies used in the present study.
