# Supplementary material for: Essential Role of Adhesion GPCR, GPR123, for Human Pluripotent Stem Cells and Reprogramming towards Pluripotency
Source: Cells. 2023 Jan 13;12(2):304. doi: 10.3390/cells12020304 (PMC9856511; doi:10.3390/cells12020304)
Supplement: Supplementary file 1 [file cells-12-00304-s001.zip › cells-2128346-supplementary.pdf]

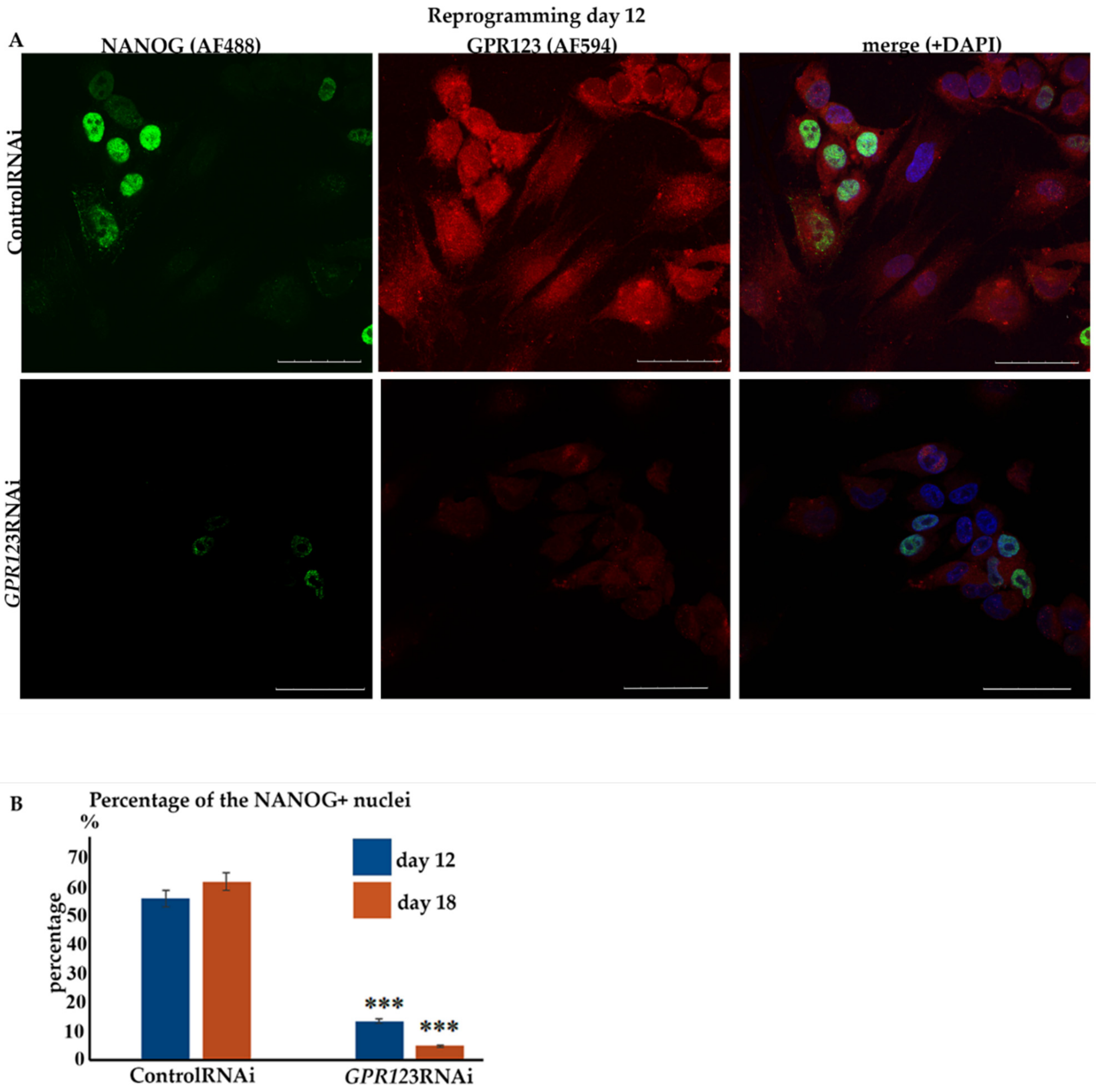

Reprogramming day 12
A
NANOG (AF488)
GPR123 (AF594)
merge (+DAPI)
ControlRNAi
GPR123RNAi
B
Percentage of the NANOG+ nuclei
%
percentage
70
60
50
40
30
20
10
0
day 12
day 18
***
***
ControlRNAi
GPR123RNAi

Figure S1. Downregulation of GPR123 significantly reduced the percentage of NANOG positive cells during the reprogramming process. (A): Representative Confocal immunofluorescence images of the NANOG and GPR123 staining in the Control (upper panel) and *GPR123*RNAi (bottom panel) hiPSCs at day 12 of the reprogramming process. Scale bar 50 µm. (B): Graphical representation of the percentage of the NANOG+ nucleuses in Control and *GPR123*RNAi treated hiPSCs at day 12 of the reprogramming. At least 100 colonies were included in the analysis during tree independent experiments.

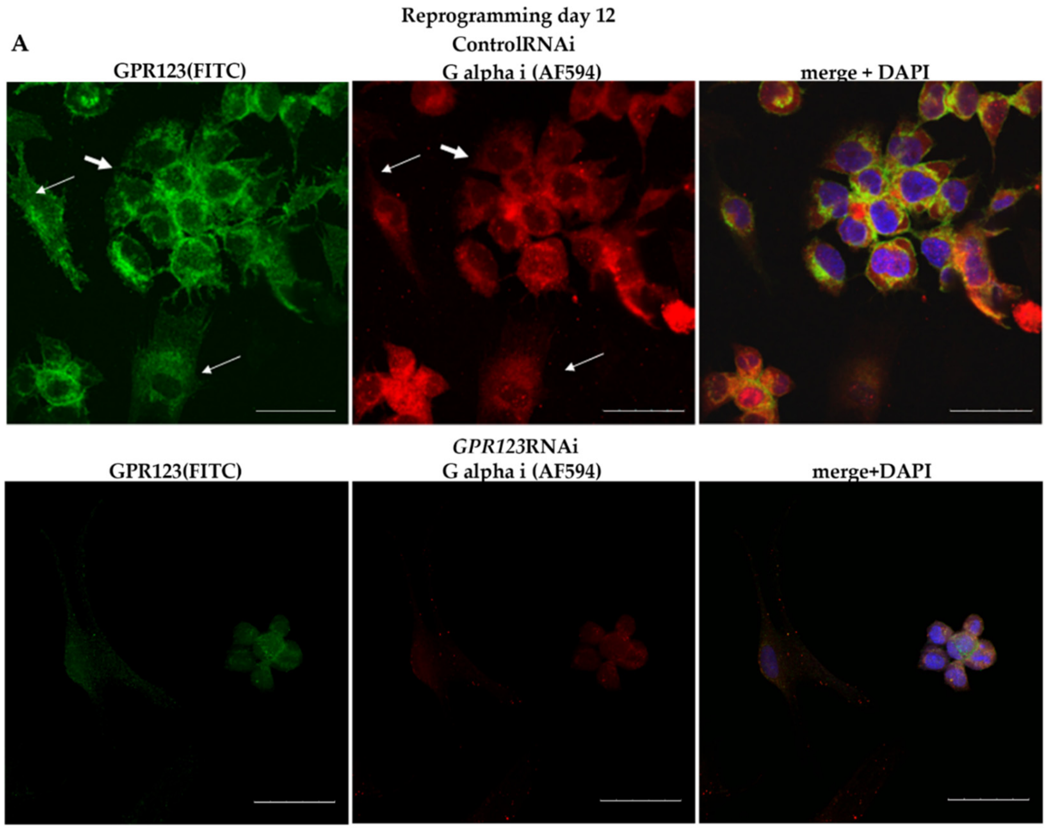
Reprogramming day 12
A
ControlRNAi
GPR123(FITC)
G alpha i (AF594)
merge + DAPI
GPR123RNAi
GPR123(FITC)
G alpha i (AF594)
merge+DAPI

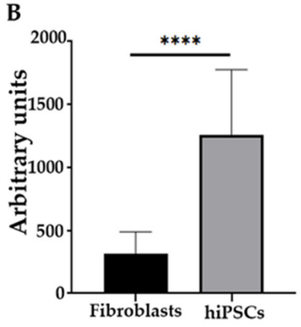
B
2000
1500
1000
500
0
Arbitrary units
****
Fibroblasts
hiPSCs

Figure S2. Co-localization between Gαi and GPR123 is lost under *GPR123*RNAi in hiPSCs at day 12 of the reprogramming process. (A): Representative Confocal immunofluorescence images for Gαi and GPR123 in hiPSCs treated with Control (upper panel) and *GPR123*RNAi (bottom panel) at day 12 of the reprogramming. Scale bar 50 µm. (B): GPR123 (FITC) mean fluorescence intensity difference between fibroblasts and hiPSCs calculated in arbitrary units using ImageJ. Bars and error bars represent normalized means and s.d. (n = 25 for each cell types; t-test, P<0.0001).

Table S1. List of the primers for qRT-PCR used in the present study.

Table S1. List of the primers for qRT-PCR used in the present study.

	Gene	Forward Primer	Reverse Primer
GPR123	ADGRA1	GTC AAC GTC AGC AGA CTC GT	CTT CTC GGA GGT GCA CGT TA
cAMP-PKA pathway related genes	ADCY5	CAT TGA TAG GCT TCG GTC TG	CGA TGG TGA TCT GGA CAA AG
	PRKAR1A	GGG AGC TTT GGA GAA CTT GC	GCT TTC TCA GTG TGC TTC CC
G-protein subunit alpha genes	GNAS	CGA CGA CAC TCC CGT CAA C	CCC GGA GAG GGT ACT TTT CCT
	GNAI1	TTAGGGCTATGGGGAGGTTGA	GGTACTCTCGGGATCTGTTGAAA
	GNAI2	TACCGGGCGGTTGTCTACA	GGGTCGGCAAAGTCGATCTG
	GNAI3	ATCGACCGCAACTTACGGG	AGTCAATCTTTAGCCGTCCCA
	GNAQ	TGGGTCAGGATACTCTGATGAAG	TGTGCATGAGCCTTATTGTGC
	GNA11	GGCTTCACCAAGCTCGTCTAC	CACTGACGTACTGATGCTCG
	GNAZ	GGTCCCGGAGAATTGACCG	ATGAGGGGCTTGTACTCCTTG
	GNAO1	GGAGCAAGGCGATTGAGAAAA	GGCTTGTACTGTTTCACGTCT
	GNA12	CCGCGAGTTCGACCAGAAG	TGATGCCAGAATCCCTCCAGA
	GNA13	CAGCAACGCAAGTCCAAGGA	CCAGCACCCTCATACCTTTGA
	GNA14	GAGCGATGGACACGCTAAGG	TCCTGTCGTAACACTCCTGGA
	GNA15	CCAGGACCCCTATAAAGTGACC	GCTGAATCGAGCAGGTGGAAT
Cell cycle-related genes	CCNB1	AAC TTT CGC CTG AGC CTA TTT T	TTG GTC TGA CTG CTT GCT CTT
	CCNA	CAT GGA CCT TCA CCA GAC CT	TCT GGT GGG TTG AGG AGA G
	CCNE	CCC CAA AGT CAC AGT CGA AG	GGG GAG AGG AGA AGC CCT AT
	CCND1	GGC GGA GGA GAA CAA ACA G	AGG CGG TAG TAG GAC AGG A
	CCND2	GGG GAG AGG AGA AGC CCT AT	ATA TCC CGC ACG TCT GTA GG

	CCND3	CAT GTA CCC GCC ATC CAT	AGC TTC GAT CTG CTC CTG AC
	CDC25A	ACT TTG TCT GAT GAG GAT GAT GG	GGT TGT CAA GGT TTG TAG TTC TC
	CDC25B	AGA CAG TAG ACG GAA AGC AC	GCT CTT CAG TAG GAA GCT CTC
	CDC25C	GTC GCT ATC CAT ATG AGT ATC TGG	GTC CTC TTC ACG CAG ACA G
Apoptosis related genes	BAX	GAC GGC AAC TTC AAC TGG	AAG TCC AAT GTC CAG CCC
	BAD	TGA GTG ACG AGT TTG TGG AC	CAA GTT CCG ATC CCA CCA
	BCL2L11	GGC CCC TAC CTC CCT ACA	GGG GTT TGT GTT GAT TTG TCA
	BCL2	GCCTTCTTTGAGTTCGGTG	GCC AGG AGA AAT CAA ACA GAG
	BCLXL	CCAGGGACAGCATATCAGAG	CAG TGT CTG GTC ATT TCC GA
	XIAP	GGCAGATTATGAAGCACGG	CCAATCAGTTAGCCCTCCTC
Pluripotency marker genes	MYC	TCG CAA GAC TCC AGC GCC TT	GGA GCC CCA ATT AAA GGC G
	OCT4	GAG AAC CGA GTG AGA GGC AAC C	CAT AGT CGC TGC TTG ATC GCT TG
	NANOG	AAT ACC TCA GCC TCC AGC AGA TG	TGC GTC ACA CCA TTG CTA TTC TTC
	SOX2	TTG TTC GAT CCC AAC TTT CC	ACA TGG ATT CTC GGC AGA CT
	KLF4	TTA CCA AGA GCT CAT GCC ACC	GCG AAT TTC CAT CCA CAG CC
Ectoderm marker genes	SOX1	GGA ATG GGA GGA CAG GAT TT	ACT TTT ATT TCT CGG CCC GT
	NESTIN	GAG AGG GAG GAC AAA GTC CC	CCA CTT CCT CAG ACT GCT CC
	PAX6	AGT TCT TCG CAA CCT GGC TA	TGG TAT TCT CTC CCC CTC CT
Mesoderm/ mesendoderm marker genes	MIXL	GAG ACT TGG CAC GCC TGT	GGT ACC CCG ACA TCC ACT T
	HAND1	ACC AGC TAC ATC GCC TAC CTG ATG	TCC CTA TTA ACG CCG CTC CAT
	MSX2	TGG ATG CAG GAA CCC GG	AGG GCT CAT ATG TCT TGG CG
	T	CAG TGG CAG TCT CAG GTT AAG AAG GA	CGC TAC TGC AGG TGT GAG CAA
	FOXA2	GCA TTC CCA ATC TTG ACA CGG TGA	GCC CTT GCA GCC AGA ATA CAC ATT
Endoderm marker genes	CDX2	CTC GGC AGC CAA GTG AAA AC	CTC CTT TGC TCT GCG GTT CT
	SOX17	CGC ACG GAA TTT GAA CAG TA	GGA TCA GGG ACC TGT CAC AC
	GATA4	TCC AAA CCA GAA AAC GGA AG	AAG GCT CTC ACT GCC TGA AG
	VIM	GAGAACTTTGCCGTTGAAGC	GCT TCC TGT AGG TGG CAA TC
	EOMES	CCA GGT TCT GGC TTC CGT G	TAC ATT TTG TTG CCC TGC ATG TT
MET-related genes	CDH1	TGC CCA GAA AAT GAA AAA GG	GTG TAT GTG GCA ATG CGT TC
	CDH2	ACA GTG GCC ACC TAC AAA GG	CCG AGA TGG GGT TGA TAA TG

	ZEB1	CCA CAC GAC CAC AGA TAC GG	CCT GAG GAG AAC TGG TTG CC
	TWIST1	CCT CCC TGT CAG ATG AGG AC	TCT GGA GGA CCT GGT AGA GG
	SNAI1	CCT CCC TGT CAG ATG AGG AC	CCA GGC TGA GGT ATT CCT TG
	SNAI2	GGG GAG AAG CCT TTT TCT TG	TCC TCA TGT TTG TGC AGG AG
Housekeeping genes	GAPDH	TGC ACC ACC AAC TGC TTA GC	GGC ATG GAC TGT GGT CAT GAG
	RN18S	GAA ACT GCG AAT GGC TCA TTA A	GAA TTA CCA CAG TTA TCC AAG TAG GA
	RPL13A	CCT GGA GGA GAA GAG GAA AGA GA	TTG AGG ACC TCT GTG TAT TTG TCA A

cells MDPI

Table S2. List of the primary antibodies used in the present study.

Target	Source	Cat. No
anti-GPR123	GeneTex, US	GTX55266
anti-OCT4	Cell Signaling Technology	2890
anti-Nanog	ThermoFisher Scientific	PA1-097X
anti-Vimentin	Cell Signaling Technology	5741
anti-E-cadherin	Cell Signaling Technology	3195
anti-N-cadherin	BD Biosciences, US	610921
anti-cyclin D1	Santa Cruz	sc-246
anti-cyclin E	Santa Cruz	sc-481
anti-cyclin B1	Santa Cruz	sc-752
anti-cyclin A1	Santa Cruz	sc-7852
anti-p-CREB (Ser133)	Cell Signaling Technology	9198
anti-p-ERK(Thr202/Tyr204)	Cell Signaling Technology	4370
anti-p-FAK (Tyr397)	Cell Signaling Technology	8556
anti-β-actin	Santa Cruz	sc-130656
anti-GAPDH	Cell Signaling Technology	2118
